# Triple-wide-band Ultra-thin Metasheet for transmission polarization conversion

**DOI:** 10.1038/s41598-020-65881-6

**Published:** 2020-06-01

**Authors:** Ayesha Kosar Fahad, CunJun Ruan, Shafaat A. K. M. Ali, Rabia Nazir, Tanveer Ul Haq, Shahid Ullah, Wenlong He

**Affiliations:** 1 School of Electronics and Information Engineering, Beihang University, Beijing, China; 2 Beijing Key Laboratory for Microwave Sensing and Security Applications, Beihang University, Beijing, 100191 China; 3 NED University of Engineering & Technology, Karachi, Pakistan; 4 University of Engineering & Technology, Lahore, Pakistan; 5 College of Electronics and Information Engineering, Shenzhen University, Shenzhen, China

**Keywords:** Electrical and electronic engineering, Electronic devices

## Abstract

Polarization converters play an important role in modern communication systems, but their wide and multiple band operation to facilitate volume and size reduction is quite challenging. In this paper, a triple-band Linear Polarization to Circular Polarization (LP-to-CP) converter is presented using a novel design procedure based on geometric parameters optimization of a metasheet. The proposed converter is ultrathin, wideband, stable over a wide range of incident angles, and polarization diverse. The conductor layer of metasheet is patterned with a square ring and five square-patches diagonally intersecting each other. To validate the proposed method, an LP-to-CP convertor in X-band (7.3~9.6 GHz) and dual Ka-bands (25.4~31.4 GHz, 35.4~42.2 GHz) is presented. The performance is quite stable in wide range of frequencies and against the variation of incident angles from −25° to 25°. After performing model-based theoretical paradigm analysis, and full-wave simulation and optimization, the converter is fabricated and the measurements are performed inside the anechoic chamber. The measured results, close to simulation results, depict the validity and reliability of the proposed design.

## Introduction

The ever increasing demand for high-speed and high-capacity communication prompts advancements in 5^th^ generation (5 G) and 6^th^ Generation (6 G) wireless communication. Polarization is an important property of the electromagnetic field which describes the direction in which electric field oscillates while the wave passes through the media. In order to control the electromagnetic waves, manipulation of this polarization state has gained much attention in the last decade due to its potential applications in stealth, satellite communication, cloaking and applications requiring asymmetric transmission characteristics^1–3^. In the microwave and mmWave range, polarization mismatch, atmospheric absorption and multi-path fading degrade the performance of the channel. In such a scenario, CP waves are preferred over LP waves because of their lower sensitivity towards the multi-path fading. In order to obtain CP waves, there can be two ways: one way is to generate them using CP antenna or use LP wave generated by an antenna and convert it into CP wave using LP-to-CP converter.

Multiband CP waves can be obtained from the CP antenna elements using regular methods such as multi-feed antennas, helical antennas and spiral antennas^4^. Multiband CP waves can also be obtained using shared aperture antennas^5,6^. However, they are difficult to design at high frequencies because of their complex feeding structures. An efficient solution is to use multi-band LP-to-CP conversion in cascade with LP antenna. There has been an increasing trend towards multiband and multifunctional polarization conversion devices^7–9^ so that multiple systems may be merged and subsequently efficient miniaturization may be achieved. An LP-to-CP converter can broadly be categorized into a reflection-based and a transmission-based converter. Reflection based converter tends to block the feeding signals. So, transmission-based converters are being investigated for the potential applications requiring incident and outgoing waves to be aligned.

Periodic structures such as metamaterials and chiral metamaterials have been used due to their well-known properties such as polarization conversion and asymmetric transmission^10–12^. Metasurfaces as 2D equivalent of metamaterials, have been explored for a variety of applications including polarization manipulation^13^ and LP-to-CP conversion^14–29^. Multi-band polarization conversion is relatively a new concept. Recent research works have been reported for dual-band polarization control including cross-polarization conversion^30,31^ and LP-to-CP conversion devices^32–40^. Liu *et al*.^30^ and Huang *et al*.^31^ reported dual-band cross polarization converters using metasurfaces. Dual-band LP-to-CP conversion operation has been reported in reflection mode^32^ and transmission modes using multi-layered^33,34,36,37^ and single-layered metasurfaces^38–40^. Zhu *et al*.^40^ proposed all-dielectric metamaterial based dual-band LP-to-CP conversion at 6.24 GHz and 6.38 GHz. But, rare work has been reported for triple-band polarization conversion operation^41–45^. Liu *et al*.^41^ presented the design of reflection-based multiband polarization converter having dual-band LP-to-CP conversion and single-band cross-polarization conversion using tri-layered metasurface based structure. They proposed ‘L’ patterned structure, which converts incident linear polarization into circular polarization in 9.1~16.5 GHz and 20.0~25.4 GHz. Moreover, it acts as a cross-polarization converter from 17.4~18.9 GHz band. Yao *et al*.^42^ proposed ultra-thin reflection-based triple-band cross polarization converter in THz regime using metasurfaces. The proposed structure consists of double ‘L’ based graphene patches and converts incident linear polarization at 36.15 THz, 48.95 THz and 52.20 THz to cross-polarization. Huang *et al*.^43^ reported E-shaped metamaterial based LP-to-CP converter at 10.1 GHz, 11.7 GHz, and 14.2 GHz. M. Fartookzadeh *et al*.^44^ reported a tri-band LP-to-CP converter based on dual-layer patch arrays working in reflection modes. It converts LP wave into reflected CP wave at 2 GHz, 8 GHz and 12 GHz. All these proposed tri-band polarization converters^41–45^ were based on reflection modes. Such converters tend to block the feeding signals and increase the overall profile for the antenna systems. Although, significant advancement has been made in dual-band transmission-based polarization converters^33–39^, triple-band transmission-based polarization converter is still a challenging problem. In our previous work^45^, we numerically proposed a triple-band LP-to-CP converter in THz regime, but operating bandwidths were narrow.

In this paper, the realization of a triple-wide-band LP-to-CP converter using a new design procedure based on geometric parameters optimization is presented. The proposed converter has five unusual properties. First, it performs LP-to-CP transmission conversion in three bands, which is practically reported the first time to the best of our knowledge. Secondly, since the structure is based on a single dielectric layer, it makes it an ultrathin device. Thirdly, it works over 19% to 20% bandwidth for all the tree bands of operation which is the widest among other reported multiband transmission based polarization conversion devices. Fourthly, the structure is low-cost and maintains performance over ±25° change in incidence angles. Lastly, the outgoing circular polarizations in second and third bands are orthogonal to the first band which makes it a good candidate for applications requiring polarization diversity.

## Results

### Unit cell design

Generally, any electromagnetic wave transmitted from a metasurface has two electric field components. One has the same polarization as that of the incident wave while the other has opposite polarization. They are called co-polarized and cross-polarized components respectively. For polarization manipulating devices, cross-coupling between transmitted electric and magnetic fields interacts with each other to produce co and cross-polarized components. For multi-band polarization conversion, coupling between electric and magnetic fields should be deliberately controlled to have multiple Eigen modes. Single-band LP-to-CP conversion has been achieved using many diagonal symmetric/semi-symmetric anisotropic structures^21–24^. Dual-band LP-to-CP operation has been achieved using center-connected^35^ and diagonal symmetric^30^ structures. Here, we selected a diagonal symmetric structure, with multiple square patches to have different resonance characteristics so that multiband operation may be realized.

**Figure 1:**
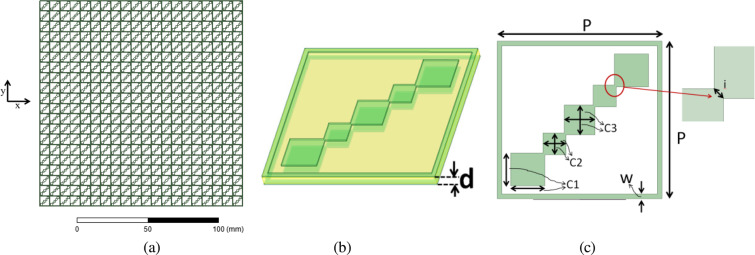
Schematic of the design: (a) Two-dimensional (2D) periodic array structure (b) 3D perspective view (c) Top view (x-y).

The proposed structure is shown in Fig. 1(a) whose unit cell is constructed by periodic unit cells shown in Fig. 1(b),(c). It consists of two identical layers of conductor patterns based on penta-squares arranged diagonally inside a square ring. Metallic layers are separated by one substrate layer. For the design parameters, center frequencies of three bands of interest are defined as: ‘${{\rm{f}}}_{1}$’, ‘${{\rm{f}}}_{2}$’ and ‘${{\rm{f}}}_{3}$’. As an example, converter’s design is presented for LP-to-CP operation at 8.5 GHz, 28.5 GHz and 38.8 GHz for a geostationary satellite. So, f_1_ = 8.5 GHz, f_2_ = 28.5 GHz, f_3_ = 38.8 GHz. Arithmetic mean of three frequency points denoted as ‘${\rm{f}}$’, can be calculated as (${{\rm{f}}}_{1}+{{\rm{f}}}_{2}+{{\rm{f}}}_{3})/3$ which equals 25.26 GHz. Thickness for the substrate was chosen to be *d* $={\rm{\lambda }}/40$, ${\rm{\lambda }}$ being the free-space wavelength at ${\rm{f}}$. Design steps were performed in standard electromagnetic software package High Frequency Structure Simulator (HFSS) using master-slave boundary conditions. Floquet ports at the input and output of the unit cell were applied to realize periodic array structure. FR4 having a thickness as 0.3 mm and relative permittivity as 4.3 and loss tangent as 0.03 was used as a substrate.

**Figure 2:**
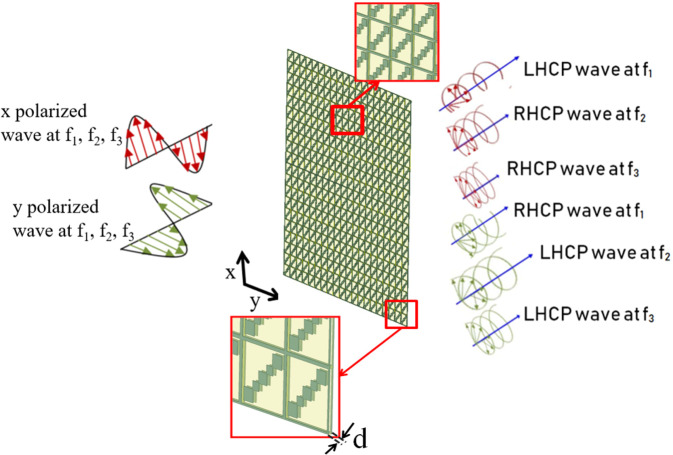
Scheme of metasheet for triple-wide-band centered at f_1_, f_2_, and f_3_.

**Figure 3:**
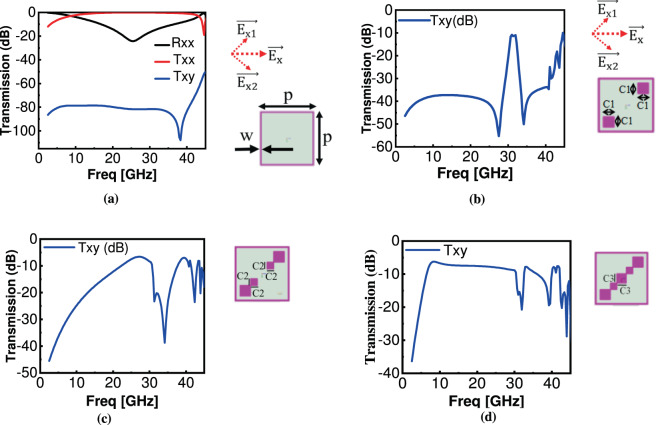
Optimization of the unit cell to achieve polarization conversion (**a**) Unit cell with square ring alone (**b**) square ring with two diagonal square patches 2xC1 (**c**) square ring with four diagonal square patches 2xC1, 2xC2 (**d**) square ring with five diagonal square patches 2xC1, 2xC2, C3.

Triple band LP-to-CP conversion scheme using metasheet scheme with orthogonal outgoing polarizations is shown in Fig. 2. For incident x-polarized waves, transmitted waves behave as LHCP at f_1_ and RHCP at f_2_ and f_3_. Whereas for incident y-polarized waves, transmitted waves behave as RHCP at f_1_ and LHCP at f_2_ and f_3_. Following are the detailed design steps for metasheet:

***Step 1****:* A square ring-based metamaterial structure is proven to be a wide-band-pass filter. The first design step involves the design of a wideband filter covering all three required frequency points. For this, a square ring with length ‘PxP’ and width ‘w’ was simulated and optimized. The structure should resonate at ‘f’ and transmission characteristics should cover all three frequency points ${{\rm{f}}}_{1}$, ${{\rm{f}}}_{2}$ and ${{\rm{f}}}_{3}.$ Transmission characteristics with optimized parameters are shown in Fig. 3(a) which shows that under incident x-polarized wave, the transmitted wave is also x-polarized from 8 GHz to 42 GHz with no polarization conversion as Txy<−80 dB. The reason for no cross-polarization conversion is symmetricity of square ring along x and y axes. In order to understand this, lets resolve incident wave $\overrightarrow{{{\rm{E}}}_{{\rm{x}}}}$ into its two orthogonal components lying at +45° and −45° depicted in Fig. 3(a) as $\overrightarrow{{{\rm{E}}}_{{\rm{x}}1}}$ and $\overrightarrow{{{\rm{E}}}_{{\rm{x}}2}}$. Since these orthogonal components will experience the same structure while transmitting through the metasurface, so no cross-polarization conversion will take place. As polarization conversion requires a different phase response for incident wave along its orthogonal components^8^.

***Step 2:*** In the next step, an-isotropicity is introduced to the unit cell in 3(a) so that polarization conversion takes place. This step involves the design of a square C1xC1 which is placed along the diagonal with corner to corner distance from step 1’s square ring as 2*w. It is clear from Fig. 3(b) that with the addition of a small an-isotropicity, cross-polarization component Txy increases. Dimensions of C1 should be chosen in such a way to have Txy peak at f_23_ where f_23_ = (f_2_ + f_3_)/2 = 33.65 GHz. It is clear from Fig. 3(b) that cross-polarization conversion occurs around (f_2_+f_3_)/2. Introduced an-isotropicity (two diagonal squares) makes unit cell asymmetric. So, orthogonal components of incident wave lying at +45° and −45°, $\overrightarrow{{{\rm{E}}}_{{\rm{x}}1}}$ and $\overrightarrow{{{\rm{E}}}_{{\rm{x}}2}}$ will experience different structures, resulting in different transmitted amplitude and phase responses. This time $\overrightarrow{{{\rm{E}}}_{{\rm{x}}1}}$ will experience extra square patches along its path characterized as inductances in series with capacitance. Through optimization in EM-simulator, dimensions of C1xC1 can be set in such a way to obtain 90° phase shift for $\overrightarrow{{{\rm{E}}}_{{\rm{x}}1}}$ at f_23_.These transmitted components will interact with each other to have transmitted y-polarized component resulting in cross-polarization conversion.

***Step 3:*** The third step involves the design of square C2, whose position and dimensions are different than C1 and results in another band of cross-polarization component. This dual-band response is due to the different eigen modes of the unit cell. Dimensions of C1 and square ring were kept fixed as found out from step 2 and C2 was optimized to get two transmission curves around f_2_ and f_3_. Amplitude response is as shown in Fig. 3(c).

***Step 4*:** The last step involves the addition of a central square C3. The addition of this square will give rise to a new third transmission band at f_1_ due to excitation of another eigen mode. Figure 3(d) shows an amplitude response from a single-layered metasurface structure containing a square ring and five diagonal squares.

**Figure 4:**
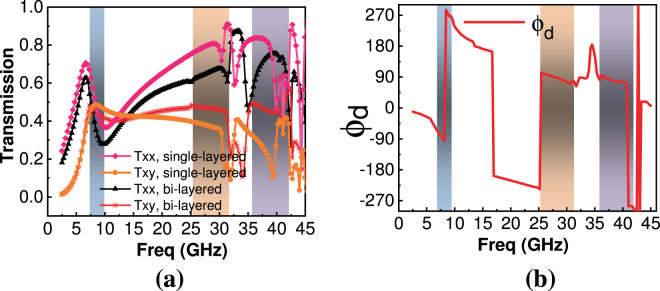
(**a**) Simulated transmission characteristics of the metasheet (**b**) Simulated phase difference between Txx and Txy.

Following the design guide, the complete unit cell shown in Fig. 1 was optimized using bi-layered structure. Ansoft HFSS’s sequential non-linear gradient programming optimizer was used in this regard. The criterion for performance of metasheet is discussed in the analysis section. A comparison between design and final optimized values is presented in Table 1. Figure 4(a) shows optimized transmission characteristics for single-layered and bi-layered metasheet. It is evident that Txy improves for bi-layered structure keeping the shape of the response nearly the same. Since the fabrication and assembly of single and bi-layered metasheet remains almost the same as the substrate is singly-layered for both. Whereas for structures having no. of metallic layers >2, fabrication, assembly and cost of the metasheet increases. So, a bi-layered structure was selected for polarization conversion to have good transmission characteristics^22^ and low-cost fabrication. Figure 4(b) depicts the difference between phases of Txx and Txy for the incident x- polarized wave respectively. Further discussion on Fig. 4(a),(b) is carried out in the Analysis section.

**Table 1:** Design and optimized parameters for the metasheet.

Parameter	Using Design Procedure (mm)	Optimized and fabricated (mm)
d	0.298	0.3
P	7.1	7.2
C1	1.48	1.5
C2	0.96	1.0
C3	1.34	1.30
w	0.2	0.2
i	—	0.05

### Analysis

For the detailed operation of a triple-band LP-to-CP converter, let’s consider a plane electric field $\overrightarrow{{E}_{xi}}$ and $\overrightarrow{{E}_{yi}}$ with x-polarization and y-polarization respectively. Considering a reflectionless ideal transmission-based polarization converter, $\overrightarrow{{E}_{xi}}$ and $\overrightarrow{{E}_{yi}}$ waves travelling in ‘+z’ direction are made incident on the surface of the converter. Corresponding outgoing waves $\overrightarrow{{E}_{xo}}$ and $\overrightarrow{{E}_{yo}}$ can be expressed in terms of transmission coefficients as shown in Eq. (1). Here, ‘$xo$’ and ‘$yo$’ represent outgoing waves corresponding to incident x- and y-polarized respectively. The equivalent matrix form representation for $\overrightarrow{{E}_{xo}}$ and $\overrightarrow{{E}_{yo}}$ can be written as:

1 $$[\begin{array}{c}{\overrightarrow{E}}_{xo}\\ {\overrightarrow{E}}_{yo}\end{array}]=[\begin{array}{cc}{\overrightarrow{t}}_{xx} & {\overrightarrow{t}}_{xy}\\ {\overrightarrow{t}}_{yx} & {\overrightarrow{t}}_{yy}\end{array}]\,[\begin{array}{c}{\overrightarrow{E}}_{xi}\\ {\overrightarrow{E}}_{yi}\end{array}]$$

Where,

2 $$\overrightarrow{{E}_{xi}}={E}_{xi}\overrightarrow{{e}_{x}}={E}_{o}{e}^{-jKz}\overrightarrow{{e}_{x}}$$

Equation (1) in terms of magnitude and phase responses, and Eq. (2) can be written as:

3 $$[\begin{array}{c}\overrightarrow{{E}_{xo}}\\ \overrightarrow{{E}_{yo}}\end{array}]=[\begin{array}{cc}|{t}_{xx}|{e}^{j{\varnothing }_{xx}} & |{t}_{xy}|{e}^{j{\varnothing }_{xy}}\\ |{t}_{yx}|{e}^{j{\varnothing }_{yx}} & |{t}_{yy}|{e}^{j{\varnothing }_{yy}}\end{array}]\,[\begin{array}{c}\overrightarrow{{E}_{xi}}\\ \overrightarrow{{E}_{yi}}\end{array}]=[\begin{array}{cc}|{t}_{xx}|{e}^{j{\varnothing }_{xx}} & |{t}_{xy}|{e}^{j{\varnothing }_{xy}}\\ |{t}_{yx}|{e}^{j{\varnothing }_{yx}} & |{t}_{yy}|{e}^{j{\varnothing }_{yy}}\end{array}]\,[\begin{array}{c}{E}_{o}{e}^{-jKz}\overrightarrow{{e}_{x}}\\ {E}_{o}{e}^{-jKz}\overrightarrow{{e}_{y}}\end{array}]$$

Where, ${t}_{xx}$ and ${t}_{xy}$ are transmission coefficients for transmitted x- and y-polarized waves and ${\varnothing }_{xx},\,{\varnothing }_{xy}$ are phases for transmitted x- and y-polarized waves respectively under incident x-polarized wave. Similarly ${t}_{yx},{\varnothing }_{yx}$ and ${t}_{yy},{\varnothing }_{yy}$ are transmission coefficients and phases for transmitted x- and y-polarized waves for incident y-polarized wave respectively. Transmission coefficients $|{t}_{xx}|,|{t}_{xy}|$, $|{t}_{yx}|$, $|{t}_{yy}|$ can be computed as:

4 $$|{t}_{xx}|=\frac{|{E}_{xo}|}{|{E}_{xi}|},\,|{t}_{xy}|=\frac{|{E}_{yo}|}{|{E}_{xi}|},\,|{t}_{yx}|=\frac{|{E}_{xo}|}{|{E}_{yi}|},\,|{t}_{yy}|=\frac{|{E}_{yo}|}{|{E}_{yi}|}$$

For the sake of simplicity, let us consider one case when incident x-polarized wave strikes the proposed device. Owing to the fact that the structure of the device is made anisotropic deliberately, the magnitudes and phases for x-polarized (${t}_{xx},{\varnothing }_{xx}$) and y-polarized transmitted waves (${t}_{xy},{\varnothing }_{xy}$) may be different. However, if for a certain frequency range these transmission coefficients become comparable and the difference between their phase angles becomes ±90°, i-e $|{t}_{xx}|$≈$|{t}_{xy}|$ and ${\varnothing }_{d}={\varnothing }_{xx}-{\varnothing }_{xy}=2n\pi \pm \pi /2$, where $n=0,\,\pm \,1,\,\pm \,2\ldots$ is an integer. This would be the condition where x-polarized wave will be converted into a CP wave. To describe the transmission conversion performance for the proposed structure, we calculate axial ratio (AR) for the transmitted wave in Eq. (5) ^33^:

5 $$AR={\left(\frac{|{t}_{xx}{|}^{2}+{|{t}_{xy}|}^{2}+\sqrt{a}}{|{t}_{xx}{|}^{2}+{|{t}_{xy}|}^{2}-\sqrt{a}}\right)}^{1/2}$$

Whereas ‘*a*’ can be calculated from Eq. (6) as:

6 $$a=|{t}_{xx}{|}^{4}+{|{t}_{xy}|}^{4}+2|{t}_{xx}{|}^{2}|{t}_{xy}{|}^{2}\,\cos (2{\varnothing }_{{\rm{d}}})$$

**Figure 5:**
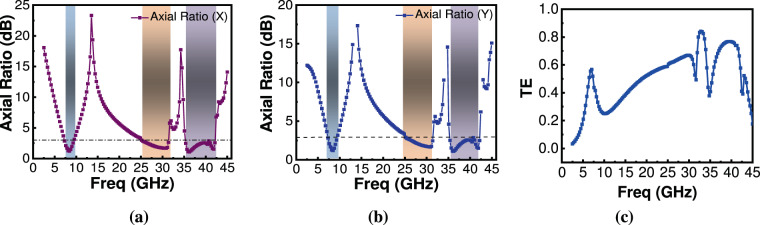
Simulated transmitted wave characteristics (**a**) AR for x-polarization (**b**)AR for y-polarization (**c**) TE.

For an ideal LP-to-CP converter, $|{{\rm{t}}}_{{\rm{xx}}}|$ = $|{{\rm{t}}}_{{\rm{xy}}}|$ and ${\varnothing }_{{\rm{d}}}={\varnothing }_{{\rm{xx}}}-{\varnothing }_{{\rm{xy}}}=2{\rm{n}}{\rm{\pi }}\pm {\rm{\pi }}/2$. In such a scenario, the outgoing wave is perfectly CP wave and AR will be 1 (0 dB). However, in most of the communication systems, 3 dB value of AR is acceptable^32–40^. Figure 4(a),(b) show that in frequency bands from 7.3~96 GHz, 25.4~31.4 GHz and 35.4~42.2 GHz the transmission coefficient magnitudes for bi-layered metasheet are comparable whereas phase difference between them is around 90° or +270°. Thus, the condition for LP-to-CP conversion is fully met at some frequencies within these bands. Though, there is some difference between $|{{\rm{t}}}_{{\rm{xx}}}|,|{{\rm{t}}}_{{\rm{xy}}}|$ particularly in the second band of operation (25.4~31.4 GHz) and at an inner frequency range of 35.4~42.2 GHz, but the difference in their phase angles ${\varnothing }_{{\rm{d}}}$ is around 90°. So, it is partially fulfilling the requirement (in this case transmitted wave will be slightly elliptical polarized). But, the performance criterion for linear to circular transmission type conversion (AR within 3 dB) is maintained. Figure 5(a) shows the response of calculated AR for the transmitted wave under incident x- polarization. It is clear that from 7.3~9.6 GHz, 25.4~31.4 GHz and 35.4~42.2 GHz AR is lower than 3 dB. So, the transmitted wave is considered to be a CP wave. Furthermore, Fig. 4(b) shows, in the frequency range from 7.3~9.6 GHz, ${\varnothing }_{{\rm{d}}}$ is around +270° which is in fact −90°, so it can be deduced that y-component of the transmitted wave is ahead of the x-component hence transmitted wave is LHCP, whereas in frequency range 25.4~31.4 GHz and 35.4~42.2 GHz, ${\varnothing }_{{\rm{d}}}$ is around +90° which means y-component of the transmitted wave is lagging behind x-component, hence transmitted wave is RHCP in these bands.

In addition, the proposed unit cell behaves equally well for the incident y-polarized wave. Figure 5(b) shows AR for the transmitted wave under incident y-polarization. However, due to the change in phase response (not shown here), the type of polarization changes in all three frequency bands resulting in RHCP for 7.3~9.6 GHz band and LHCP for 25.4~31.4 GHz and 35.4~42.2 GHz frequency bands. From this property of the proposed structure, it can be inferred that if it is required to have opposite types of circular polarization, one should simply rotate the structure to 90°. Transmission efficiency (TE) of the converter which is defined as ${\rm{TE}}=|{{\rm{t}}}_{{\rm{yx}}}{|}^{2}+|{{\rm{t}}}_{{\rm{xx}}}{|}^{2}$ is as shown in Fig. 5(c). Although transmission performance in the first band is not as good as in the second and third bands reaching worst value of 25% at 9.6 GHz, it reaches best value of 52.1%, 67%, and 77% in three bands of operation which is comparable with other single and dual- band converters^27–29,40^.

**Figure 6:**
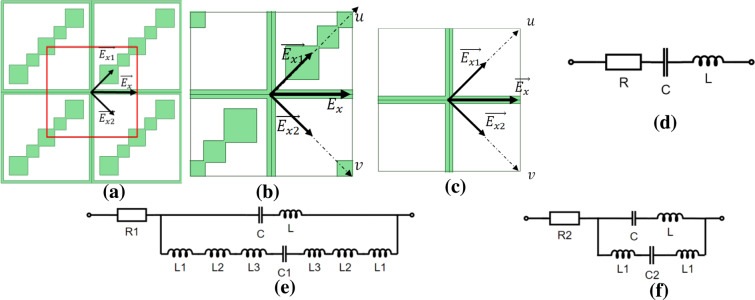
(**a**) Four unit cells for the proposed metasheet (**b**) New unit cell (**c**) New unit cell with diagonal patches removed (**d**) Equivalent Circuits for unit cell with diagonal patches removed (**e**) An equivalent circuit for the new unit cell as seen by $\overrightarrow{{E}_{x1}}$. (**f**) An equivalent circuit for the unit cell as seen by $\overrightarrow{{E}_{x2}}$.

Here we use the equivalent circuit model as adopted in^46^ for the proposed metasheet to show how it can convert incident LP wave into a CP wave. For this, we redraw the proposed metasheet as shown in Fig. 6(a) with a new unit cell shown in red and enlarged as in Fig. 6(b). Moreover, we have introduced a new coordinate system which is 45° rotated from xy coordinate system, denoted as uv coordinate system. Let’s consider that incident wave is x-polarized wave represented as $\overrightarrow{{E}_{x}}$. We can break it into its two orthogonal components as $\overrightarrow{{E}_{x1}}\,{\rm{and}}\,\overrightarrow{{E}_{x2}}$ lying along u and v axes respectively. It is important to mention here that new unit cells presented in Fig. 6(b),(c) are symmetric w.r.t u and v axis, so no cross-polarization conversion will happen for incident u and v polarized waves^8^. We have removed the diagonal patches in Fig. 6(c) to make the structure a symmetric one which is essentially a symmetric wire-grid based metasurface whose impedance model under $\overrightarrow{{E}_{x1}}$ and $\overrightarrow{\,{E}_{x2}}$ is as shown in Fig. 6(d) and can be given as in Eq. (7):

7 $$Z=R+j(wL-\frac{1}{wC})$$

Where, R and L are the equivalent resistance and inductance of the metallic strips and C is the capacitance formed by $\overrightarrow{{E}_{x1}}$ or $\overrightarrow{{E}_{x2}}$ on the adjacent metallic orthogonal strips. For the new unit cell in Fig. 6(b), impedance model under $\overrightarrow{{E}_{x1}}$ is as shown in Fig. 6(e). Metallic diagonal patch along $\overrightarrow{{E}_{x1}}$ formed by square patches can be considered as inductances corresponding to each square in series with capacitance. We have presented equivalent resistance for Fig. 6(b) as seen by $\overrightarrow{{E}_{x1}}$ as R1. $\overrightarrow{{E}_{x2}}$ will see the new unit cell as equivalent circuit as shown in Fig. 6(f) formed by capacitance in series with inductances. New impedances for Fig. 6(e),(f) can be written as shown in Eqs. (8) and (9):

8 $$Z1=R2+\left(jwL-\frac{j}{wC}\right)||\left(2jw(L1+L2+L3)-\frac{j}{wC1}\right)$$

9 $$Z2=R1+\left(jwL-\frac{j}{wC}\right)||\left(2jwL1-\frac{j}{wC2}\right)\,$$

The resultant phase difference between transmitted waves along u and v axes will be ang (Z1)-ang(Z2). Since the proposed metasheet is based on multiple sized and differently spaced square patches, Z1 and Z2 will have different values in different frequency bands. If the metasheet is designed in such a way that |Z1| = |Z2| and ang (Z1)-ang (Z2) = ±90° for all the three frequency bands. The resultant outgoing wave will be circularly polarized.

**Figure 7:**
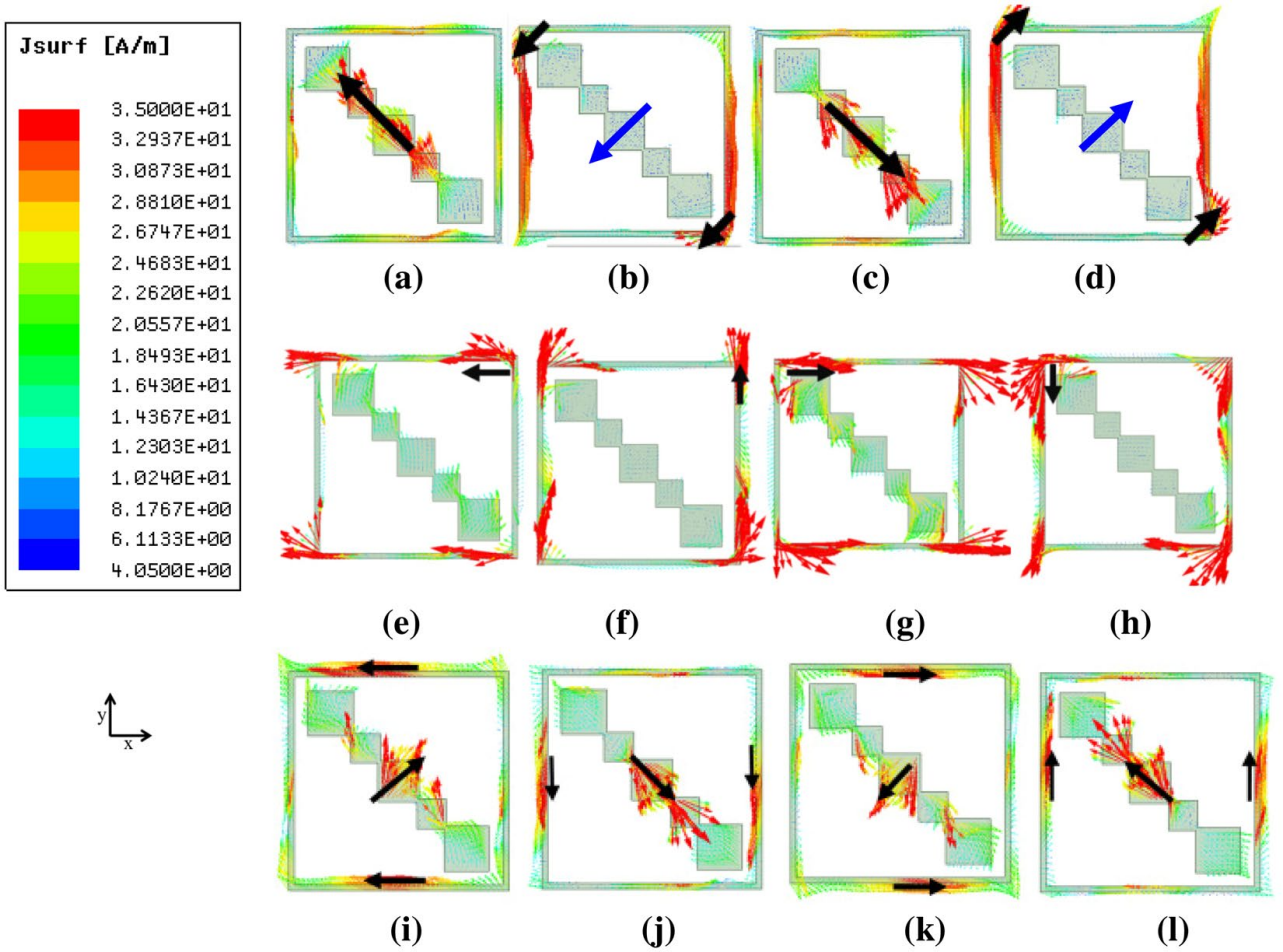
Surface current distribution of the proposed multi-band metasheet at (**a**) f_1_, t = 0, (**b**) f_1_, t = T/4 (**c**) f_1_, t = T/2 (**d**) f_1_, 3 T/4 (**e**) f_2_, t = 0 (**f**) f_2_, t = T/4 (**g**) f_2_, t = T/2 (**h**) f_2_, 3 T/4 **(i)** f_3_, t = 0 (**j**)f_3_, t = T/4 **(k**) f_3_, t = T/2 (**l**) f_3_, 3 T/4.

To explain the physical phenomenon behind the proposed LP-to-CP operation, we considered the surface current vectors at the outgoing surface of the unit cell at frequency points in each band, let it be f_1_, f_2_, f_3_ with values as f_1_ = 8.45 GHz, f_2_ = 30.83 GHz, f_3_ = 40 GHz. Figure 7 shows the surface current distribution for f_1_, f_2_, f_3_ under the incident x-polarized wave. It is important to mention here that the surface current distribution is at the transmitting surface of the proposed converter for different time intervals. Here ‘T’ represents the time period for the incident wave at the corresponding frequency. It can be seen in Fig. 7(a) that at t = 0, surface current vectors are making an angle of 135° with the +x axis. For the ease of understanding, we have shown the strength of current vectors at the center for Fig. 7(b),(d) by a blue arrow. Figure 7(b) shows that at t = T/4, the surface current vectors are making an angle 225° with the +x axis. At t = T/2, the angle changes to 315°. For t = 3 T/4, this angle reaches 45°. Thus, with every time T/4, surface current vectors rotate with an angle 90° in a counter-clockwise direction. So, the transmitted wave is LHCP at f_1_ which is in accordance with Fig. 4(b) (y-component is ahead of the x-component). Figure 7(e–l) show the surface current vectors at the output surface at f_2_ and f_3_ respectively at different time intervals. It can be clearly seen that with every quarter cycle, surface currents are rotated 90° in a clockwise rotation hence transmitted wave is RHCP at f_2_ and f_3_. This rotation agrees to phase difference in Fig. 4(b) (y-component lags x-component).

## Experiment

**Figure 8:**
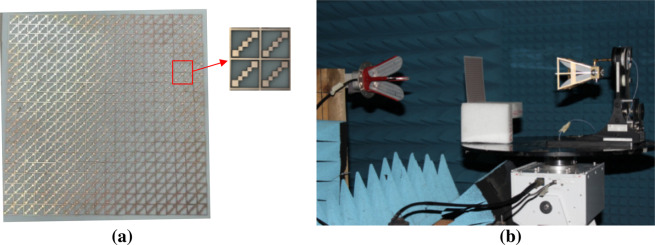
(**a**) Photograph of the fabricated metasheet (**b**) Test setup for triple-wide-band metasheet in transmission mode.

In order to validate the design and simulation procedure, the proposed metasheet for triple-band LP-to-CP conversion was fabricated using a normal PCB fabrication procedure. The size of the sample (20 × 20 elements) was 144 × 144 mm^2^. Figure 8(a) shows the photograph for the fabricated sample. Testing was carried out in two sets due to broadband coverage of the metasheet in X and Ka bands. For this, two sets of horn antennas were used. The first set was used to perform testing from the frequency range of 1~18 GHz, while the second set was used to test the converter from 26.5~40 GHz frequency range. Radiation waves from the transmitting horn antennas were x-polarized. The line of sight between both sets of horn antennas passed through the center of metasheet. Antennas were placed at a suitable distance from device to ensure uniform plane wave strikes the device. Testing was performed inside the anechoic chamber. Rohde & Schwarz ZVA-50 was used for the measurement of transmission parameters. The test setup is as shown in Fig. 8(b). Two horn antennas were connected to the Vector Network Analyzer (VNA) in the control room of the anechoic chamber via long coaxial cables. They acted as transmitting and receiving antennas. Orientation for the receiver antenna was changed for x- and y-polarized waves.

**Figure 9:**
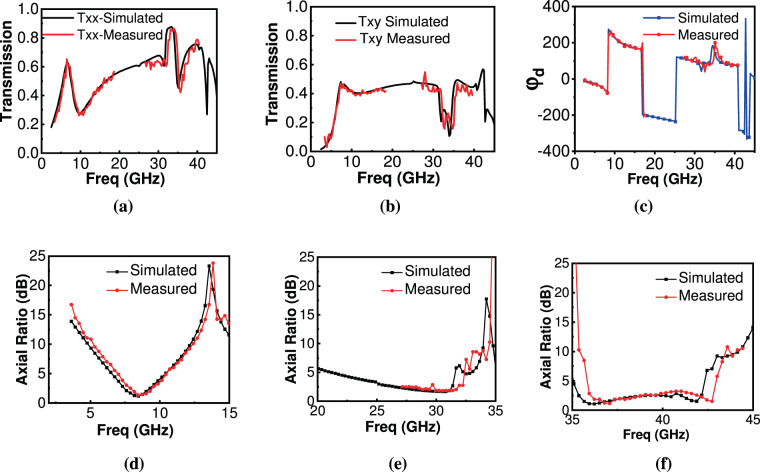
Simulated and measured response for metasheet (**a**) Txx (**b**) Txy (**c**) ${\varnothing }_{{\boldsymbol{d}}}$ (**d**) AR in X-band (**e**) AR in first Ka-band band (**f**) AR in second Ka-band.

After choosing a suitable distance between antennas and metasheet, the sample was removed for the reference measurement of amplitude and phase response. For this $|{{\rm{t}}}_{{\rm{xx}}}|$, $|{{\rm{t}}}_{{\rm{xy}}}|$ with respective phases were measured. Then, the metasheet was placed in between the two antennas to measure $|{{\rm{t}}}_{{\rm{xx}}}|$ and $|{{\rm{t}}}_{{\rm{xy}}}|$ with their phases. Final transmission coefficients and their phases were calculated by subtracting from the reference measurement. In this way, losses due to the test environment and test cables were mitigated. Figure 9(a),(b) depict measured and simulated responses for $|{{\rm{t}}}_{{\rm{xx}}}|$ and $|{{\rm{t}}}_{{\rm{xy}}}|$ respectively. It is clear that both measured results agree to simulated results, though there is a mild frequency shift in a higher band of operation. Measured and simulated difference between transmitted waves’ phases is presented in Fig. 9(c).

Figure 9(d)–(f) show measured and simulated curves for AR response for the transmitted wave for X and dual-Ka-band respectively. It is evident from Fig. 9(d) that metasheet performs well in X-band, with a very close agreement between measured and simulated results. For the first Ka-band operation, the performance in the lower band could not be tested but it is expected to be as in the higher frequency band. Figure 9(e) shows that although measured results are agreed well with the simulation results, a small glitch in AR around 29.5 GHz was observed but it remained lower than 3 dB. Also, from Fig. 9(f), it is clear that for the third frequency band, AR reached 2.5 dB around 40 GHz. The performance of metasheet remained quite well in the third band too, except the frequency shift. These variations in AR for all three frequency bands might be due to the fabrication tolerance and non-ideal conditions of the test setup. Ka-band is the most sensitive and has a larger frequency shift. The overall performance of the metasheet has a good agreement with the simulated results.

**Figure 10:**
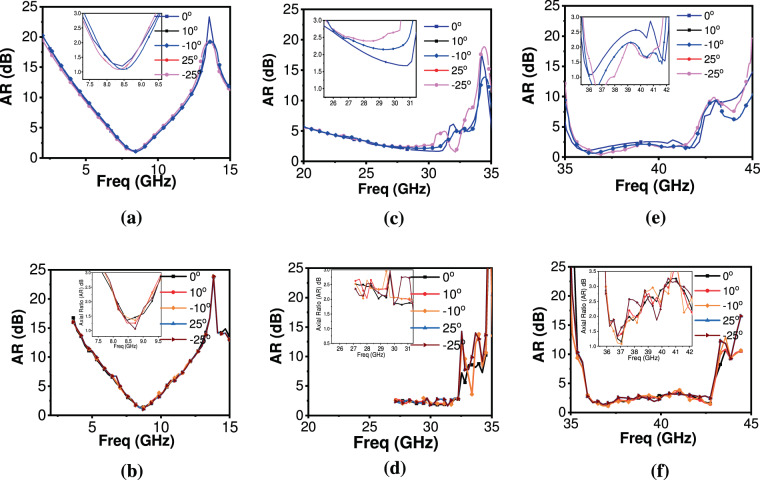
The response of metasheet under different oblique incidences (**a**) Simulated AR in X-band (**b**) measured AR in X-band (**c**) Simulated AR in first Ka-band, (**d**) Measured AR in first Ka-band, (**e**) Simulated AR in second Ka-band, (**f**) Measured AR in second Ka-band.

The performance of metasheet in all three frequency bands under variations in incident angles was simulated, analyzed and is presented in Fig. 10. It can be inferred that metasheet maintains performance under different incident angles except for some discrepancies found in higher frequency end of the second band. It is important to mention here that due to the unavailability of wideband horn antennas, frequency bands 18 GHz to 26.5 GHz and 40 GHz to 46 GHz remained untested. However, close agreement between test results and simulated results from 1~18 GHz, and 26.5~40 GHz ensures the design validity for the proposed ultrathin metasheet.

Table 2 presents the performance comparison of the proposed metasheet with other state-of-the-art multiband LP-to-CP converters. It can be inferred that the proposed metasheet performs over a wide range of frequencies in three bands of operation. Besides, the structure is ultra-thin with a minimum number of metallic layers. It maintains angular stability over −25°~+25°variation in the incident angle.

**Table 2:** Performance comparison among other transmission type multiband LP-to-CP converters.

	Centre Frequencies (GHz)	AR bandwidth	Thickness	Cell size	Metallic layers	Polarization modes	Angular stability
^33^	Dual band (19.95, 29.75)	2.5%, 1.7%	0.07λ_1_	0.35λ_1_	3	Orth	30°
^36^	Dual band(19.6, 29.6)	4%, 2.7%	0.31λ_1_	—	5	same	—
^37^	Dual band (7.6, 13)	31.6%, 13.8%	0.24λ_1_	—	4	same	±25°
^40^	Dual band(18.5, 29)	29%, 12%	0.1λ_1_	0.25λ_1_	2	orth	20°
This work	Triple band (8.45, 28.4, 38.8)	27.2%, 21.2%, 17.5%	0.008λ_1_	0.2λ_1_	2	orth	±25°

*λ_1_ is free space wavelength at centre frequency of first band of operation.

## Conclusion

In summary, a triple-wide-band LP-to-CP converter is realized based on ultrathin transmissive metasheet using step by step design procedure. The operating principle for linear to triple-wide-band polarization conversion was analyzed in detail. Numerical and surface current analyses were carried out to verify the behavior of outgoing wave in X-band (7.3~9.6 GHz), and dual Ka-bands (25.4~31.4 GHz, 35.4~42.2 GHz) bands with 27.2%, 21.2% and 17.5% operating bandwidths respectively. With incident x(y) polarized wave, the proposed metasheet transmits LHCP (RHCP) in X-band and RHCP (LHCP) in dual Ka-bands. In order to validate the performance, a prototype was fabricated and tested inside anechoic chamber using two sets of standard antennas. Experimental results have a reasonable agreement with the simulations. The behavior of proposed metasheet with the change in incident angle was investigated numerically and experimentally, and it was verified that with the change in incident angle from −25° to +25°, the response remained almost stable. The proposed ultrathin, dual-polarized metasheet may have potential applications in polarization-manipulation devices. Moreover, it opens new horizons for future wireless communication systems including satellite communication systems for volume and size reduction.

## Methods

### Simulation

Electromagnetic simulation was carried out by Ansoft HFSS which is based on Finite Elemenet Mesh (FEM) Method. The unit cell was defined along x and y directions whereas its thickness was modeled along z-axis. Periodic boundary conditions (master-slave) were applied at x- and y- faces of unit cell. Electromagnetic waves were made incident at the one face of the unit cell along +z axis, while transmitted wave’s components were observed from the other side of the metasheet. FR4 substrate was modeled with relative permittivity as 4.3 and slightly higher dielectric loss tangent as 0.03 considering the performance requirement in Ka-band.

### Fabrication and measurement

Metasheet consisted of 20 × 20 elements was fabricated using standard printed circuit board technology. Rohde & Schwarz ZVA-50 was used for the measurement of transmission parameters inside the anechoic chamber. Standard double ridged wideband horn antenna for 1~18 GHz measurement and Ka-band antennas with focusing lense for 26.5~40 GHz measurement range were used. Dielectric focusing lense was used for measurement in Ka-bands. Reference measurements were subtracted from the sample’s measurements. The receive antenna was rotated 90°to measure the cross-polarization component. For measurements under different incident angles, the transmit antenna was rotated clockwise and anti clock-wise for measuring transmission performance with ±10°, ±25° incident angles.

## Data Availability

Supporting data for the presented study is available from the corresponding author upon request.
